# Effect of enzymatic treatment on *Eucalyptus globulus* vessels passivation

**DOI:** 10.1038/s41598-023-29861-w

**Published:** 2023-02-17

**Authors:** Álvaro Vaz, João Coelho, Vera Costa, Thaddeus Maloney, Josphat Phiri, Paula Pinto, António Mendes de Sousa, Rogério Simões

**Affiliations:** 1 Fiber Materials and Environmental Technologies (FibEnTech-UBI), Universidade da Beira Interior, R. Marquês de D’Ávila e Bolama, 6201-001 Covilhã̃, Portugal; 2 Department of Bioproducts and Biosystems, School of Chemical Engineering, Aalto University, 02150 Espoo, Finland; 3 RAIZ-Forestry and Paper Research Institute, Qta. S. Francisco, Apartado 15, Eixo, 3801-501 Aveiro, Portugal

**Keywords:** Materials chemistry, Chemical engineering

## Abstract

Hardwood vessel elements generate problems in industrial uncoated wood-free printing paper operation, causing vessel picking and ink refusal. These problems are mitigated using mechanical refining at the cost of paper quality. Vessel enzymatic passivation, altering its adhesion to the fiber network and reducing its hydrophobicity is a way of improving paper quality. The object of this paper is to study how the enzymatic treatment by xylanase and by an enzymatic cocktail containing cellulases and laccases affect elemental chlorine free bleached *Eucalyptus globulus* vessel and fiber porosities, bulk, and surface chemical compositions. Thermoporosimetry revealed the vessel structure to be more porous, surface analysis showed its lower O/C ratio and bulk chemistry analysis its higher hemicellulose content. Enzymes had different effects on porosity, bulk and surface composition of fibers and vessels, affecting vessel adhesion and hydrophobicity. Vessel picking count decreased 76% for papers containing vessels treated with xylanase and 94% for the papers with vessels treated with the enzymatic cocktail. Fiber sheet samples had lower water contact angle (54.1º) than vessels rich sheets (63.7º), that reduced with xylanase (62.1º) and cocktail (58.4º). It is proposed that differences in vessel and fiber porosity structures affect the enzymatic attacks, eventually causing vessel passivation.

## Introduction

Hardwoods are more advanced and complex in their anatomical organization than softwoods, including many different cell types, such as fibers, vessels, and parenchyma. Vessel elements are the specialized water-conducting cells of hardwoods^1^, being the main conducting element in hardwoods, and its cells have entirely open or perforated ends. The cell walls of vessels are comparatively thin, and are highly ornamented with pits^2,3^. Vessels represent an important volumetric percentage of eucalypt wood, which can vary between 10 to 30%, corresponding to 3 to 5% of the weight of eucalypt^4^. Morphologically, their length can vary between 200 and 600 µm and their width can be greater than 500 µm; their width/length ratio vary between 1:1 and 1:3^5^. Vessel specific characteristics and their behavior in the uncoated wood free (UWF) paper production process remains poorly understood^6^. Some of the main problems associated with vessel elements arise when UWF paper is printed, namely vessel picking and ink refusal.

The vessel picking phenomenon manifests itself as hardwood vessel elements detach from the paper surface in offset printing due to their weak bonding and to the printing ink high viscosity^7^. The number of vessel elements picked off during printing depends on the number, size, and shape of vessel elements in the paper surface, on the bonding strength between vessel elements and the paper sheet, and on the number and bonding strength of fibers covering the vessel elements^3,7^. Thus, the reduction of vessel picking tendency may be achieved by reducing the vessel content with a suitable hardwood raw material selection, removing large vessel elements with hydrocyclones, reducing the size of vessel elements by mechanical action, such as refining, increasing vessel to fiber bonding strength by increasing the conformability of fibers, using pulp with high hemicellulose content, forming a suitable sheet structure, and subjecting the pulp to chemical or enzymatic action.

Although increased refining action can eventually increase vessel fibrillation and flexibility, it can also result in poor drainage. So enzymatic treatment has been object of study to reduce vessel picking and increase vessel adhesion. A patent of disclosure from Honshu Paper Co.^8^ describes the use of commercial cellulases to enhance the flexibility of hardwood vessels. Enzyme treatment with cellulases was shown to significantly reduce vessel picking^8,9^. Treatment of unbleached hardwood pulp with a mixture of cellulases and xylanases was found to chemically change the hardwood vessel elements, rendering them prone to break under normal mill refining^10,11^. The influence of different commercial cellulases and xylanases or their mixtures on the quality of different bleached kraft pulps was also investigated^12^.

The chemical composition of fiber surface can be significantly different from the bulk composition of the fibers^13^. X-ray Photoelectron Spectroscopy (XPS), through samples exposure to x-radiation releases electrons which allows the identification of the surface chemical composition at a depth of 1–5 µm. The effect of cooking conditions on the surface compositions of unbleached softwood *Pinus sylvestris* kraft pulp has been investigated by XPS^14^. An enrichment of alkyl carbon in the outermost surface layers was observed most probably due to lignin reprecipitation, and a strong extractives enrichment in the surface of non-extracted pulp when the alkaline charge decreased. The enrichment of lignin in the fibers surface layers of *Pinus sylvestris* kraft pulp was also observed after bleaching^15^. Elemental chlorine free (ECF) bleached kraft pulps from *Eucalyptus globulus* (*E. globulus*) and other hardwoods^16^ were investigated by detailed bulk chemical analysis, showing remarkable differences in xylan/cellulose ratios, carboxyl groups content and, particularly, in lipophilic extractives, which were assigned to differences in wood compositions, pulping and bleaching conditions. XPS analysis showed the fibers surface extractives content to be much higher than that in the inner parts of fibers.

Vessel bulk and surface chemistry, morphology, and nanostructure of vessel elements have been studied after separation of vessel elements and fibers, using eucalypt kraft pulp and recycled pulp as raw materials^17,18^. The surface morphology, surface chemical characteristics and chemistry of fibers and vessel elements were found to be similar using field emission scanning electron microscopy (FE-SEM), microbeam X-ray photo-electron spectroscopy (µ-XPS) and time-of-flight secondary ion mass spectrometry (ToF–SIMS). However, normalized lignin peak intensities of ToF–SIMS indicated that vessels had lower surface lignin counts than fibers, also containing lower bulk extractives content, but similar surface coverage. The effects of cooking and bleaching on the vessel chemistry and ultrastructure were also studied^6^. Vessel elements and fibers had a similar pyrolysis–gas chromatography–mass spectrometry (Py-GC/MS) bulk chemical composition, the content of cellulose and lignin being the most significant difference, i.e., vessel elements were richer in cellulose compared to fibers, and lignin could be found in the vessels even after bleaching stages.

The cell wall of wood exhibits porosity or micro-voids of molecular scale dimensions, due to the partial filling of space between the cellulose microfibrils by lignin, hemicelluloses, and extractives. Hemicelluloses have been shown to participate in the regulation of the nanoscale architecture of cell wall constituents, influencing the aggregation of cellulose and of the pattern of inter-unit linkages in lignin^19,20^. Understanding the geometry of the microvoids is of great importance to understand processes such as chemical pulping and bleaching, wood decay, wood-water interactions and the chemical modification of wood^21,22^. The cooking and bleaching processes, removing lignin and hemicelluloses, change the porosity of the cell wall^23^. A variety of physical techniques are available for determining the geometry of the micropores of the cell wall, such as gas sorption isotherms, mercury intrusion porosimetry, solute exclusion, thermoporosimetry, nuclear magnetic resonance and microscopic techniques^21^. Thermoporosimetry uses differential scanning calorimetry to explore the fact that water has a depressed melting temperature in small pores. Three fractions of the water inside the cell wall may be considered: freezing and nonfreezing water in relatively small pores (micropores) and bulk water in relatively large pores (macropores)^23,24^.

Understanding vessel structure regarding fiber structure is fundamental to understand chemicals access during pulping and bleaching processes and enzymes access in enzymatic treatments, and how it affects the bulk and surface chemical composition of vessels and fibers. Thus, the object of this paper is to study how the enzymatic treatment by xylanase and by an enzymatic cocktail containing cellulases and laccases affect ECF bleached *E. globulus* vessel and fiber structure, and chemical bulk and surface composition. It is proposed that differences in vessel and fiber structures affect the enzymatic attacks, which results in different bulk and surface compositions evolutions, eventually leading to vessel passivation through fiber-vessel adhesion increase and vessel hydrophobicity reduction. For that purpose, a vessel fiber separation method was implemented to obtain vessel-rich and fiber-rich suspensions, that were studied as such and after enzymatic treatment either with xylanase or with the cellulase and laccase cocktail. Fiber and vessel porosity structures were studied using thermoporosimetry and electron spectroscopy for chemical analysis/ X-ray photoelectron spectroscopy (ESCA/XPS) was used to study their surface composition. The bulk chemistry was studied for both vessels and fibers, obtaining their carbohydrate composition, total acids content, hexenuronic acids content, and ionic character using zeta potential. Vessel picking tests using IGT equipment were done on paper sheets enriched with vessels as such and treated with either xylanase or the cellulase and laccase cocktail. Water contact angle was measured on compressed fiber rich sheets and vessel rich sheets surfaces with and without enzyme treatment, to evaluate vessel comparative hydrophobicity and the enzymatic effect.

## Materials and methods

### Raw material

The pulp used in the production of fiber and vessel rich suspensions was ECF bleached *E. globulus* reference pulp provided by RAIZ—Forestry & Paper Research Institute (code Navigator E.B. Cacia 2019-FEU, of 3 April 2019). The Navigator Company *E. globulus* cultivated forests are certified by the Forest Stewardship Council (2007) and by the Program for the Endorsement of Forest Certification (2009)^25^.

### Separation method

Primary size exclusion separation was carried out using a three stages Bauer-McNett, with U.S. mesh # 30, # 50 and # 200 screens, corresponding to opening sizes respectively of 595 µm, 297 µm and 74 µm. The #30 screen retained long fibers, while #200 screen let fines pass through. A Britt Dynamic Drainage Jar was also set up according to the method earlier generically described^18^, adapted with a recirculation system. A two-step system was developed, initially for vessels accumulation within the jar and afterwards for vessels extraction. Two extracts were obtained, the fiber rich one containing practically pure fibers, while the vessel rich fraction contained around 1:1 ratio of vessels and fibers.

### Enzymatic hydrolysis

Parallel enzymatic hydrolysis treatments of the two samples (fiber rich samples and vessel rich samples) were performed with Novozymes endo-1,4-xylanase NS51121 and the Celodev commercial enzymatic cocktail Celodase-083S with cellulase and laccase activities (containing sorbitol, cellulase, laccase and 1,2-benzisothiazol-3-one). The xylanase treatment was carried out at 45 °C, with constant agitation at a sample consistency of 1%, and an enzymatic dosage of 0.01% (0.75 IU/g dry pulp, according to the safety datasheet). The treatment with the enzymatic cocktail was carried out at 40 °C, with constant agitation at a sample consistency of 4.5%, and an enzymatic dosage of 1% (10–100 IU/g dry pulp for cellulase and 0.5–5 IU/g dry pulp for laccase, according to the safety datasheet). Both sets of treatments were performed with no pH control requirement (samples pH of 7) for 60 min. To remove the enzymes, the samples were then subjected to three sequential washings in distilled water followed by centrifugations at 3000 g to recover the fibrous material.

### Thermoporosimetry

The thermoporosimetry measurements were performed on a Mettler Toledo DSC 3+ (Mettler-Toledo Intl. Inc. Instrument, USA) differential scanning calorimeter equipped with an intracooler. A 2–6 mg sample of pulp was hermitically sealed in 40 μL aluminum pans. The masses of the sealed crucibles were recorded before and after the measurements to ensure that all pans remained sealed during the measurement. After the measurements, the crucibles were punched with a needle and dried in an oven at 105 °C overnight to determine the moisture content. The temperature was first brought to –50 °C at 20 K/min causing all the freezable water in the samples to crystalize. The temperature was then increased to –0.2 °C and held constant until the melting transition was completed i.e., until all the water in the small capillaries melt. This step is essential to prevent supercooling during the subsequent recrystallization step. The temperature was then decreased at 2 K/min to –30 °C. In the next step, the temperature was increased at 20 K/min to + 50 °C and the total freezable water in the sample was calculated by integrating the resulting peak. The nonfreezing water (NFW) was then determined from the difference between the moisture content and the total amount of freezable water.

### Thermal gravimetric analysis

Thermal gravimetric analysis (TGA) was made on fiber rich and vessel rich samples using the Netzsch STA 449 F3 Jupiter thermogravimetric analyzer in the temperature interval of 40–1200 °C at a heating rate of 10 K/min and under a flow of air (50 ml/min) in open alumina crucibles. The weight of the samples was between 10 and 15 mg. After the experiment, the residual ash was collected and analyzed using scanning electron microscopy (SEM).

### Bulk carbohydrate/neutral sugars composition

The two fractions (fiber rich and vessel rich) were hydrolyzed following the laboratory analytical procedure for the determination of structural carbohydrates and lignin in biomass, from the National Renewable Energy Laboratory (NREL)^26^. Concisely, 300 mg of sample was successively submitted to a 72% (m/m) and 3%(m/m) sulfuric acid hydrolysis during 1 h at ambient temperature and 1 h at 121 °C, respectively. Neutral sugars, organic acids, and carbohydrate byproducts (furfural and hydroxymethylfurfural, HMF) in the hydrolysates were analyzed by using the high-performance liquid chromatography (HPLC) system. In this case a Rezex ROA (Phenomenex^®^) organic acid column was used, using a 0.005N sulfuric acid ultra-pure water, as eluent. The column was maintained at 60 °C, and the flow was 400 µL/min. Cellobiose, glucose, xylose, mannose, galactose, and arabinose were used as standards. Although this column elutes xylose, mannose, and galactose at the same retention time, not enabling their separation, it has the advantage of analyzing the samples without post-treatment procedures.

### Total acidic groups content

The total acidic groups content was determined through a conductimetric titration method, adapted from the standard SCAN-CM 65:02^27^. Firstly, each sample was protonated in a 0.1 M HCl solution. The samples were repeatedly washed by sequential resuspensions in distilled water followed by 5-min centrifugations at 3000*g*. The supernatant was discarded until it attained a conductivity of less than 5 μS/cm. Then, the samples were resuspended in a 0.001 M NaCl solution at a 0.2% consistency and were afterwards titrated with 0.05 M NaOH. The titrated samples were collected by vacuum filtration and dried overnight in an oven at 105 °C for exact mass quantification. The registered conductivity throughout the titration was plotted and the total acidic groups concentrations were calculated from the intersection of the best adjusted linear tendency lines.

### Hexenuronic acid groups

Hexenuronic acid (HexA) content was determined using UV spectroscopy on the solution resulting from the selective hydrolysis of HexA in the pulp sample in mercuric chloride–sodium acetate solution^28,29^. The dry matter content of the samples was determined according to the ISO standard 638 (2008). 0.05 g of air-dried samples were accurately weighted. 10 mL of a hydrolysis solution consisting of 2.2 mM mercuric chloride in 0.7% sodium acetate was added to the samples in glass sealed vials. The hydrolysis was carried out in a water bath at 65 °C with constant agitation. The samples were afterwards cooled to room temperature, filtered with a 0.45 μm nylon syringe filter and then measured by a spectrophotometer Spectronic Helios Gamma UV–Vis Spectrophotometer at 260 and 291 nm wavelengths against a hydrolysis solution blank. The hexenuronic acid content was calculated according to the following equation:

$$C_{HexA} = \, 0.273 \times \frac{{(A_{260} - A_{291} ) \times V}}{w}$$

where C_HexA_ is the hexenuronic acid content in the sample (μmol/g); 0.273 is a calibration factor for a standard pulp; A_260_ and A_291_ are the absorbances registered at 260 and 291 nm wavelengths, respectively; V is the hydrolysis solution added volume (mL) and w is the o.d. mass of the sample (g).

### Scanning electron microscopy and EDX elemental analysis

A vessel rich and a fiber rich aqueous suspension with solid contents of 0.1 wt% were sonicated for 5 min to improve dispersion. A few drops were placed on microscope sample holders and were air dried overnight at room temperature. Microscopy observations were performed using a Hitachi S-2700 scanning electron microscope (SEM) operated at 20 kV. Images were formed through secondary electrons. All the samples were previously gold sputtered by cathodic spraying (Quorum Q150R ES). Elemental analysis of the samples was performed using Energy-Dispersive X-ray spectroscopy (EDX) using the same SEM equipment to estimate silica, carbon, and oxygen contents. Images of the residual ash were also taken using Zeiss Sigma VP scanning electron microscopy at 5 kV. Before the analysis, the samples were coated with a gold–palladium film.

### Zeta potential

The Zeta potential was measured using a Zetasizer Nano ZS instrument (Malvern Instruments Ltd.) at 0.1% consistency. The Zeta potentials of the fiber rich and the vessel rich aqueous suspensions were also measured using a Mütek SZP-06 System Zeta Potential (Noviprofibre, France) at a 0.2% consistency. KCl was added to the suspensions to reach the minimum conductivity required to perform the measurements.

### X-ray photoelectron spectroscopy (XPS)

The surface chemical composition of the samples was analyzed with an X-ray photoelectron spectroscopy, using an AXIS Ultra spectrometer with monochromatic Al Kα irradiation source at 100 W. Before the measurements, the samples were evacuated overnight. Survey scans as well as high-resolution regional carbon and oxygen were acquired from 2 to 3 locations.

### Vessel picking characterization

The IGT Reprotest (Amsterdam, The Netherlands) testing system was used to characterize vessel picking; the system consists of an ink distribution system (IGT Reprotest B.V. Inking unit AE) and a printing apparatus (IGT Reprotest B.V. AIC2-5 printability tester). The printing tests were carried out with inks of medium viscosity and increasing printing speed (from 0 up to 6 m/s). An optical/digital method was developed to quantify the picked-up vessels in the printing tests. First, samples with the same size were scanned with an HP ENVY 4520 scanner system, generating images with a resolution of 1200 dots per inch (dpi). The scanned images were then binarized to black and white images using a FOTOR software and were afterwards processed and analyzed with ImageJ© version 1.53j, after setting appropriate size and circularity parameters. The software counted the white spots on the image, which corresponded to the vessel picks.

### Contact angle measurement

The contact angle between a drop of water and the sheet surface was measured at room temperature, using an OCAH 200, DataPhysics Instruments GmbH, (Filderstadt, Germany), with the sessile drop method. Drops of uniform size (2 to 5 μl) of distilled water were deposited on fiber rich compressed sheets and vessel rich compressed sheets surfaces and the mean contact angle was measured. The contact angle was calculated as the mean of six measurements.

### Ethics approval and consent to participate

All authors give the ethics approval and consent to participate in the article.

### Ethics declaration

All methods were carried out in accordance with relevant guidelines in the methods section.

### Plant materials research guidelines and legislation

All relevant institutional, national, and international guidelines and legislation experimental research and field studies on plants (either cultivated or wild), including the collection of plant material, were complied with all raw materials used in this study. The pulp used in the production of fiber and vessel rich suspensions was ECF bleached *E. globulus* reference pulp provided with all due licenses by RAIZ—Forestry & Paper Research Institute with the code “Navigator E.B. Cacia 2019-FEU”, of 3 April 2019. The Navigator Company *E. globulus* cultivated forests used for pulp production are certified by the Forest Stewardship Council (2007) and by the Program for the Endorsement of Forest Certification (2009)^25^.

## Results and discussion

### Thermoporosimetry

**Figure 1:**
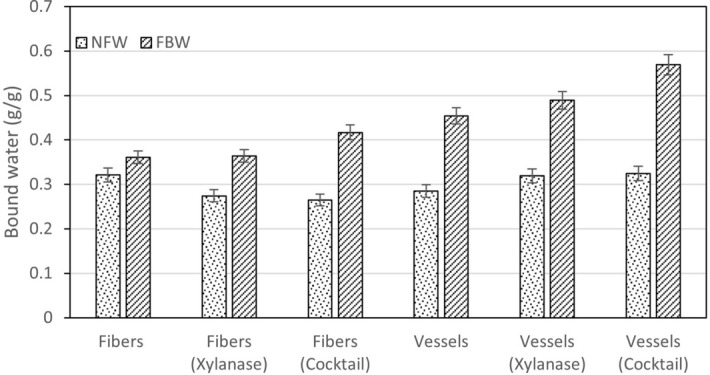
Freezing bound water (FBW) and non-freezing bound water (NFW).

**Figure 2:**
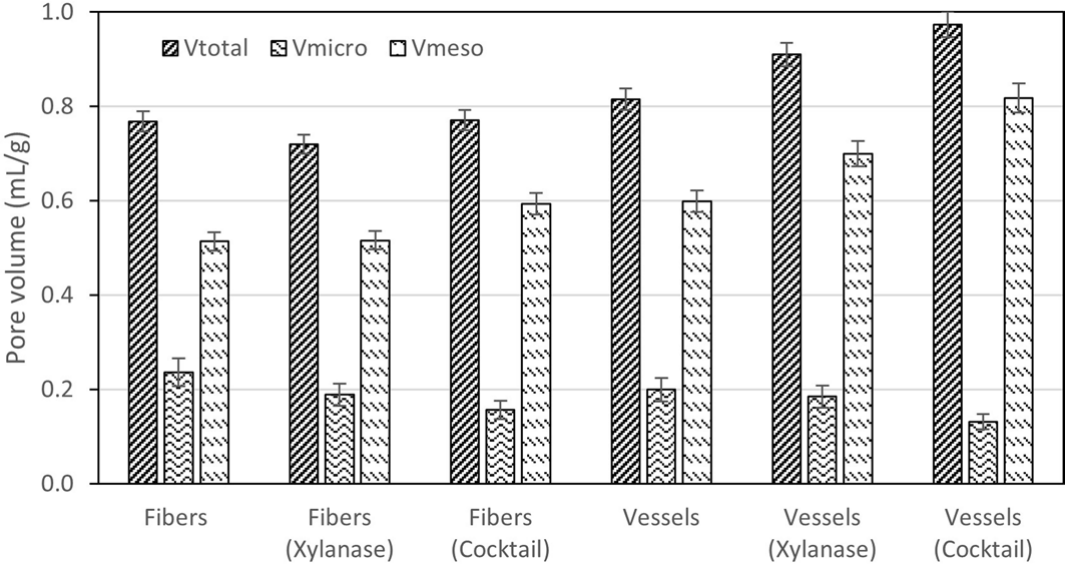
Pore composition volumes for fibers and vessels, treated and untreated.

In thermoporosimetry, differential scanning calorimetry is used to measure the amount of hydration water in pulp fibers, measuring the energy absorbed when the water in a frozen sample of pulp fibers is melted. Assuming fiber pores to be cylindrical with radius *r* Gibbs-Thompson equation calculates the pore size using the melting point and melting point depression of water at normal pressure, the specific molar volume of ice, and water surface tension.^23,24^. Pores can be classified into micropores (< 2 nm), mesopores (2–50 nm) and macropores which are above 50 nm as it is done by the IUPAC system^30,31^. According to its behavior during the differential scanning calorimetry process, water may be classified in *unbound water* whose transition temperature, enthalpy and peaks are equal to those of pure water, *bound water* (FBW) with a transition temperature lower than that of pure water, and *non-freezing water* (NFW) as a kind of bound water whose transition is not detected^32^. The thermoporosimetry results in both fibers and vessels before and after enzymatic treatment are compiled in Table 1, namely non-freezing water (NFW), freezing bound water (FBW), pore total volume (V_total_), micropores volume (V_micro_) and mesopores volume (V_meso_). Figure 1 represents freezing and non-freezing bound water for untreated and enzymatically treated vessels and fibers, while Fig. 2 shows the pore composition volumes for untreated and enzymatically treated fibers and vessels.

**Table 1:** Thermoporosimetry results in fibers and vessels, treated and untreated.

	NFW, g/g	FBW, g/g	V_total_, mL/g	V_micro_, mL/g	V_meso_, mL/g
Fibers	0.321	0.361	0.768	0.237	0.514
Vessels	0.285	0.454	0.815	0.200	0.599
Vessel/fiber Comparison	−11.2%	25.8%	6.1%	−15.6%	16.5%
Fibers (xylanase)	0.274	0.364	0.72	0.189	0.516
Fiber xylanase effect	−14.6%	0.8%	−6.3%	−20.3%	0.4%
Vessels (xylanase)	0.319	0.489	0.91	0.185	0.7
Vessel xylanase effect	11.9%	7.7%	11.7%	−7.5%	16.9%
Fibers (cocktail)	0.265	0.417	0.771	0.157	0.594
Fiber cocktail effect	−17.4%	15.5%	0.4%	−33.8%	15.6%
Vessels (cocktail)	0.324	0.569	0.974	0.132	0.818
Vessel cocktail effect	13.7%	25.3%	19.5%	−34.0%	36.6%

We can observe that vessels have less non-freezing water than fibers (−11.2%) and more freezing bound water (+ 25.8%). This signifies that the total vessel porosity is 6.1% higher than that of fibers, vessels having a lower microporosity (−15.6%) and a higher content of mesopores (+ 16.5%). Both enzymes decreased the non-freezing water in fibers while increasing in that of the vessels (fibers decrease −14.6% with xylanase and −17.4% with the enzymatic cocktail, while vessels increase 11.9% with xylanase and 13.7% with the enzymatic cocktail). Freezing bound water, by its side, for fibers has a small increase (+ 0.8%) in xylanase and a significant increase for the enzymatic cocktail (+ 15.5%); otherwise, freezing bound water has a comparatively greater increase in vessels (+ 7.7% for xylanase, and 25.3% for the enzymatic cocktail). In terms of porosity structure, fibers decrease total porosity with xylanase (−6.3%) and increase marginally (+ 0.4%) with the cocktail; fibers microporosity decrease significantly with both enzymes (−20.3% with xylanase, −33.8% with the cocktail); while mesopores have a small increase with xylanase (+ 0.4%) and a greater increase with the cocktail (+ 15.6%). Vessels by its side increase total porosity both with xylanase (+ 11.7%) and with the enzymatic cocktail (+ 19.5%) with the cocktail; vessels microporosity decrease with both enzymes (−7.5% with xylanase, −34.0% with the cocktail); mesopores volume also increase with xylanase (+ 16.9%) and with the enzymatic cocktail (+ 36.6%). Both enzymes seem to have a more significant effect on vessels than on fibers in terms of porosity structure, more pronounced in the case of the enzymatic cocktail comparatively to xylanase (as observed in Fig. 2). The effect of the enzymatic cocktail on the hemicellulose removal, as shown latter, is also more drastic than that of xylanase. Fibers have no significant alteration of its total porosity, even though microporosity reduces significantly, especially with the cocktail. This may be explained by further fibers collapse. Vessels, on the other hand, have significant total porosity increase at the expenses of micropores decrease and mesopores increase, situation more visible with the enzymatic cocktail in comparison to xylanase.

### Bulk sugars composition and acidic groups content

Table 2 compiles the values for the hemicellulose content (percentage of xylose, mannose, and galactose, referred as XMG), hexenuronic and total acids contents (µmol/g) and the pulp suspension Zeta potential (mV) as measured in Mütek SZP-06 System Zeta Potential at a 0.2% consistency. Zeta potential results agree with the previous results showing that fibers are more anionic than vessels. XMG content in vessels is 35% higher than in fibers, thus having a higher hemicellulose percentage. This result agrees with previous ones reported by other authors^33,34^. Concerning the total acids content, this value is also higher in vessels (12%). The difference is even more evident for the hexenuronic acid content (71%). Considering the higher hexenuronic and total acids content in the vessels, a higher zeta potential for the vessels would be expected, however, this is not the case. Vessels lower zeta potential can be attributed to carboxylic acids neutralization due to higher vessel cations content, as the EDX results indicate higher cationic content (Na, Si, K, Ca) and some anionic (Cl) accumulation in vessels. This fact may explain the observed zeta potential reversal.

**Table 2:** Chemical analysis and zeta potential (Mütek SZP-06).

	XMG (%)	Hexenuronic acid (µmol/g)	Total acids (µmol/g)	Zeta potential (mV)
Fibers	18.8 ± 0.2	7.0 ± 0.9	133 ± 4	−20.8 ± 0.9
Vessels	25.4 ± 0.2	12.0 ± 1.5	149 ± 4	−9.8 ± 0.5
Fibers with xylanase	18.8 ± 0.2	–	–	−24.5 ± 0.7
Vessels with xylanase	23.9 ± 0.2	–	–	–
Fibers with cocktail	15.3 ± 0.2	1.9 ± 0.5	–	−24.0 ± 0.9
Vessels with cocktail	17.7 ± 0.2	4.9 ± 0.7	–	–

*XMG* xylose, mannose, and galactose content.

### Scanning electron microscopy and EDX analysis

**Figure 3:**
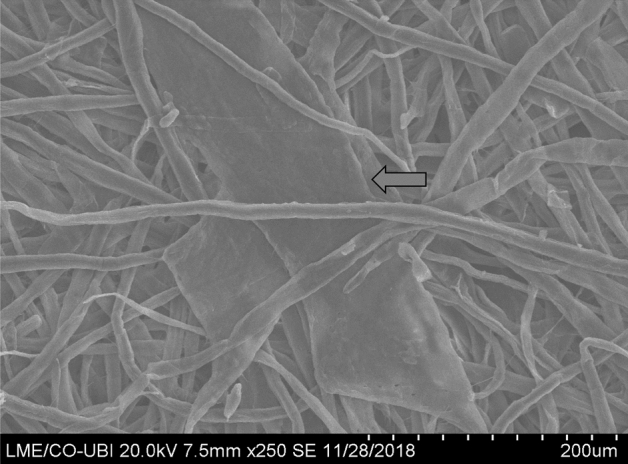
SEM image of a vessel rich sheet surface (the arrow indicates the vessels).

SEM imaging was performed on the surface of a sheet produced from the vessel rich fraction. Figure 3 depicts two noticeable vessels amongst the fibers. The EDX mass percentages results are presented in Table 3, where the most relevant result is the cationic trash (Na, Si, K, Ca) and some anionic (Cl) accumulation in vessels. The molar O/C ratios are 0.86 and 0.74 for *E. globulus* fibers and vessels respectively, suggesting higher lignin and extractives surface content for the latter.

**Table 3:** EDX mass elemental composition (%).

	C	O	Na	Al	Si	Cl	K	Ca
Fibers	46.4 ± 0.5	53.4 ± 0.6	–	0.1 ± 0.1	0.0	–	–	0.1 ± 0.0
Vessels	48.5 ± 2.8	47.8 ± 3.1	1.6 ± 1.4	–	0.9 ± 0.7	0.8 ± 0.7	0.2 ± 0.1	0.3 ± 0.1
Vessels + Xyl	49.2 ± 0.5	50.5 ± 2.6	0.2 ± 0.1	–	0.0	0.1 ± 0.0	–	0.1 ± 0.0

Concerning the oxidative TGA analysis, it was observed that silica crystal residues were obtained for the vessels, while for the fibers no residual was observed. Thus, there is a presence of silicon in untreated vessels in contrast to absence in untreated fibers. Silica bodies commonly have been observed to occur in rays and axial parenchyma cells, and acicular crystals in sheaf-like aggregates are also often found inside vessels and also yellowish-brown deposits in some vessels^35^. Silica exerts protective role initially attributed to a physical barrier fortifying the cell wall (e.g., against fungal hyphae penetration), but several studies have shown that the action of this element on plants to be far more complex, as it involves a cross-talk with the cell interior and an effect on plant metabolism^36,37^. Apparently, the treatment of vessels with xylanase decreases the amount of silicon, hinting possible binding of silicon to hemicelluloses.

### Zeta potential

Zeta potential results for untreated fibers and vessels and those submitted to enzymatic treatments are compiled in Table 4, as measured in the Zetasizer Nano ZS at 0.1% consistency. Pulp fibers are negatively charged due to the pulping procedure^38^. In unbleached kraft pulp fibers, the negative charge can be originated both from polysaccharides, with major contributions from the uronic acid groups in xylan and from lignin. The presence of acidic extractives as fatty acids may also play a role. In bleaching, some of the acidic groups can be eliminated although large differences exist between alternative bleaching sequences. Fibers in the present work have shown a stronger anionic character than vessels (−11.8 mV for fibers, −8.7 mV for vessels), in agreement with Mütek SZP-06 values. As shown previously, hemicellulose content in vessels is about 6% higher than in fibers. Therefore, higher relative charges in vessels were a priori expected, but it was not the case. Concerning enzymatic treatments, vessels further reduce their anionic character with both enzymes, in accordance with the hexenuronic acids decrease from 12.0 µmol/g to 4.9 µmol/g, as seen on Table 2. Fibers show dissimilar behaviors in both enzymes. The enzymatic cocktail reduces the anionic character in fibers, in agreement with the hexenuronic acids decrease from 7.0 µmol/g to 1.9 µmol/g. Meanwhile, xylanase applied on fibers had no significant effect on XMG content, but the zeta potential slightly increased in both Mütek SZP-06 and Zetasizer.

**Table 4:** Zeta potential (Zetasizer) for fibers and vessels, treated and untreated.

Samples	Zeta potential, mV
Fibers	−11.8 ± 0.9
Fibers cocktail	−4.5 ± 0.4
Fibers xylanase	−16.60 ± 1.3
Vessels	−8.74 ± 0.69
Vessels cocktail	−3.73 ± 0.29
Vessels xylanase	−3.57 ± 0.28

### X-ray photoelectron spectroscopy (XPS)

Table 5 compiles the XPS results for the surface relative concentrations of elements and O/C ratio for the Whatman reference and all fiber and vessel samples untreated and treated with xylanase or with the enzymatic cocktail. Table 6 compiles the relative amounts of the different carbon chemical surroundings, as compared to the total amount of carbon in the same samples. A higher surface O/C ratio was observed in fibers comparatively to vessels. This difference may arise either from lignin deposition as from extractives deposition^38^. Xylanase reduces this ratio for both fibers and vessels, while the enzymatic cocktail provokes distinct effects on fibers (3% increase) and vessels (8% decrease). Concerning the elements concentration, fibers and vessels show cellulose-like material profiles, even though most carbohydrates look like this in XPS, so it is not necessarily just cellulose. Traces of nitrogen are too low for quantification, but the element does show up in most of the data, so it may be present. The presence of nitrogen in the cocktail treated samples may suggest that residual enzymes remained adsorbed on the fibers and vessels. A significatively higher content of silica in vessels was observable. According to the vessels wide-scan, the binding energy (BE) of Si 2p is between metallic Si and SiO_2_, suggesting that Si is bonded with C and O. According to high resolution (HiRes) spectra carbon, a C–Si component seems to be present. It cannot be quantified because the BE of this component is too close to CC, but it has shifted the whole CC-component clearly to lower BE. And according to HiRes oxygen, the O1s has some O–Si nature, too. Whatever this Si–C–O component is, it looks much like being one component only, amount of which varies from spot to spot at the vessel surface. Xylanase reduces Si 2p content in both fibers and vessels, effect even more pronounced with the enzymatic cocktail. This fact in a certain way supports the possibility of a Si–C–O component. Siloxanes and phthalates are part of composition of all pulps^16,39^. Concerning the relative amount of carbon in the different forms^40,41^, the C1 peak (C–C or C–H) is due mainly to lignin and extractives. The amount of C1 decreases from extractives to lignin and finally to carbohydrates. Lignin, cellulose, and hemicellulose contribute to C2 (C–O) and C3 (C=O or O–C–O). C4 (O=C–O) peak is due to oxidized groups in cellulose and hemicellulose (and possibly lignin). As can be seen in Table 7, vessels have shown to have higher C1 content (+ 21.4%), and lower content of C2 (−2.5%), C3 (−11.2%) and C4 (−10.8%). Thus, vessels must have a higher surface content of extractives and/or lignin. Higher XPS surface coverage of extractives and lignin have been obtained elsewhere in previous works^18,42^, even though no direct support for lignin value was obtained by TOF–SIMS. The surface composition of fibers can be significantly different from the bulk composition^38^. In the case of lignin, the differences are not very large, i.e., smaller than one order of magnitude, but for the extractives the differences in composition between bulk and the surface is even larger than an order of magnitude. As previously said, fibers have shown lower extractive surface coverage than vessels^18^, even though in these same works fibers have shown higher bulk extractives. This might be explained by its higher relative surface area per unit of volume comparatively to vessels, and to the lower porosity hindering mass transfer to the surface. In what concerns to xylanase effect on carbon composition, it increased C1 content in fibers and seems to have produced an inverse effect on vessels. It also decreased C2 content in both fibers and vessels, while increasing C3 and C4. These might be the result of further release of hemicellulose and extractives, mainly in fibers. In what respects to the enzymatic cocktail, fibers decreased C1, while the opposite happened for vessels. Fibers also increased C2, decreasing C3 and C4 just marginally. Vessels, on its side, reduce C2, practically maintain C3 and significatively increase C4. These variations may also possibly be explained by release of hemicelluloses and extractives, combined with mass transport phenomena associated with porosity of the structures.

**Table 5:** Relative concentrations of elements in the samples surface (XPS).

	C 1s %	N 1s %	O 1s %	Si 2p %	O/C ratio
Ref. (Whatman)	57.04	0.00	42.96	0.00	0.753
Fibers	60.99	0.25	38.59	0.17	0.633
Vessels	61.44	0.23	37.84	0.49	0.616
Vessel fiber comparison	0.7%	−8.0%	−1.9%	188%	−2.7%
Fibers with xylanase	61.43	0.27	38.17	0.13	0.621
Fibers xylanase effect	0.7%	8.0%	−1.1%	−24%	−1.8%
Vessels with xylanase	62.06	0.33	37.29	0.32	0.601
Vessels xylanase effect	1.0%	43.5%	−1.5%	−35%	−2.4%
Fibers with cocktail	59.69	1.73	38.56	0.02	0.646
Fibers cocktail effect	−2.1%	592%	−0.1%	−88%	2.1%
Vessels with cocktail	62.18	2.19	35.45	0.19	0.570
Vessels cocktail effect	1.2%	852%	−6.3%	−61%	−7.4%

**Table 6:** Relative amounts of the different carbon chemical surroundings (XPS).

	C_1_ (C–C) %	C_2_ (C–O/C–N) %	C_3_ (C=O) %	C_4_ (O–C=O) %
Ref (Whatman)	4.55	74.59	16.98	3.88
Fibers	17.53	62.44	15.6	4.43
Vessels	21.28	60.91	13.86	3.95
Vessel/fiber comparison	21.4%	−2.5%	−11.2%	−10.8%
Fibers with xylanase	18.05	58.29	17.83	5.82
Fibers xylanase effect	3.0%	−6.6%	14.3%	31.4%
Vessels with xylanase	21.02	59.16	15.76	4.07
Vessels xylanase effect	−1.2%	−2.9%	13.7%	3.0%
Fibers with cocktail	14.99	65.58	15.02	4.40
Fibers cocktail effect	−14.5%	5.0%	−3.7%	−0.7%
Vessels with cocktail	22.74	57.34	13.93	5.99
Vessels cocktail effect	6.9%	−5.9%	0.5%	51.6%

**Table 7:** Fibers and vessels (treated and untreated) water contact angle.

Samples	Contact angle (°)
Fibers	54.1 ± 2.4
Vessels	63.7 ± 2.4
Vessels xylanase	62.1 ± 3.1
Vessels cocktail	58.4 ± 2.1

### Vessel picking and contact angle

To analyze the enzyme effects on vessel picking more clearly, vessels previously isolated from the integral pulp were added to the pulp. This pulp was previously beaten to a refining degree of 28ºSR and thereafter vessel rich fractions without and with enzymatic treatment were added to the pulp. The final vessel content of the pulp was about 3–5%. When an equivalent mass of vessels pre-treated with xylanase or the enzymatic cocktail were added to the beaten pulp, vessel picking was seen to substantially reduce. Picking count decreased from 161 ± 8 picks/dm^2^ with non-treated vessels, to 38 ± 2 picks/dm^2^ for the vessels treated with xylanase (−76%) and 9 ± 1 picks/dm^2^ for the ones treated with the enzymatic cocktail (−94%).

Water contact angle was measured on compressed fiber rich sheets and vessel rich sheets surfaces, with the results compiled in Table 7. Fiber sheet samples had lower water contact angle than the vessels rich sheets, supporting the idea that vessels are more hydrophobic than fibers. Xylanase reduced vessels hydrophobicity, with a further increase with the enzymatic complex containing xylanase. Thus, both enzymes reduce vessels hydrophobicity, creating the necessary conditions to minimize the ink refusal phenomena during the printing process.

## Discussion of results

The objective of this paper was to study how the enzymatic treatment by xylanase and by an enzymatic cocktail containing cellulases and laccases affect ECF bleached *E. globulus* vessel and fiber structures and their bulk and surface chemical compositions. It was proposed that the differences in vessel and fiber porosity structures affect the enzymatic attacks, resulting in different bulk and surface compositions evolution, eventually causing vessel passivation through fiber-vessel adhesion increase and vessel hydrophobicity reduction.

Concerning the comparative evaluation of porosity, the thermoporosimetry results showed that vessels had higher porosity than fibers, with a higher content of mesopores and lower microporosity. Both enzymes seem to have a more significant effect on vessels than on fibers in terms of porosity structure, more pronounced in the case of the enzymatic cocktail comparatively to xylanase. Fibers have no significant alteration of its total porosity, even though microporosity reduces significantly, especially with the cocktail. This may be explained by further fibers collapse. Vessels, on the other hand, have significant total porosity increase at the expenses of micropores decrease and mesopores increase, situation more noticeable with the enzymatic cocktail in comparison to xylanase.

Bulk hemicellulose content was shown to be significantly higher in vessels than in fibers, confirming previous results^33,34,42^. Total acids content was also shown to be higher in vessels, difference even more evident for the hexenuronic acid content.

Fibers surface composition can be significantly different from the bulk composition, being negatively charged due to the pulping procedure^38^. In the case of lignin, the differences are not very large, i.e., smaller than one order of magnitude, but for the extractives the differences in composition between bulk and the surface is even larger than an order of magnitude. In comparative terms, a higher surface O/C ratio was observed in fibers comparatively to vessels. This difference may result from lignin deposition as well as from extractives deposition, as XPS C_1_ results may suggest. Other works have shown that fibers have lower extractives surface coverage than vessels^6^, even though in these works fibers have shown higher bulk extractives. This might be explained by their higher relative surface area per unit of volume comparatively to vessels, and to the lower porosity hindering mass transfer to the surface. Xylanase reduces surface O/C ratio for both fibers and vessels, while the enzymatic cocktail causes a decrease on vessels but a reverse effect on fibers.

Attending to the study performed on surface charges, fibers have shown a stronger anionic character than vessels, although higher relative values in vessels were a priori expected considering the higher hexenuronic and total acids content in the vessels, but this was not the case. Vessels lower zeta potential can be due to carboxylic acids neutralization due to higher vessel cations content, as the EDX results indicate higher content of cationic trash (Na, Si, K, Ca) and some anionic (Cl) accumulation in vessels. Other possibility might be higher extractives surface deposition^38^. Concerning enzymatic treatments, vessels further reduce their anionic character with both enzymes, while fibers show dissimilar behaviors in both enzymes. The enzymatic cocktail reduces the anionic character in fibers, but xylanase causes an inverse effect.

These works demonstrated higher vessels hydrophobicity comparatively to fibers, as supported by differences in contact angles of sheets of untreated fibers and of vessels and after enzymatic treatment. These differences may result from the surface chemistry of vessels and fibers, with vessels containing higher surface extractives as shown by the O/C ratios and XPS C_1_ results. Xylanase reduced vessels hydrophobicity, the enzymatic complex further decreasing its value. Thus, both enzymes reduce vessels hydrophobicity, creating the necessary conditions to minimize the ink refusal phenomena during the printing process. A possible explanation for these results may be surface extractives content reduction by the enzymes.

The picking count study made with the IGT decreased substantially from sheets with non-treated vessels comparatively to the sheets with vessels treated with xylanase and further for the sheets with vessels treated with the enzymatic cocktail. This demonstrates that vessel adhesion to the fiber structure increases with xylanase, possibly due to a decrease in surface energy. The enzymatic cocktail still reinforces this adhesion, that may be explained by a fibrillation increase.

Attending to these different results, we consider that the initial proposed hypothesis is supported by this work results. The observed effects might be even higher if a pure vessel fraction could be obtained. So, differences in vessel and fiber porosity structures affect the enzymatic attacks, resulting in different bulk and surface compositions evolution, eventually causing vessel passivation through fiber-vessel adhesion increase and vessel hydrophobicity reduction.

## Conclusions

The main conclusions are:

1. Fiber and vessel porosimetry: vessels revealed to be more porous than fibers, with a higher content of mesopores and a lower content of micropores.
2. Fiber and vessel chemical bulk and surface chemistry: vessels have shown to have higher hemicellulose content, with higher total acids content and hexenuronic acids content. As revealed by ESCA/XPS analysis, vessels surface has lower O/C ratio and higher C1 carbon, which indicates higher surface coverage of extractives and/or lignin.
3. Enzymatic effect: vessel and fiber bulk chemistry showed a reduction in hemicellulose content, while its surface chemistry revealed hemicellulose and extractives surface content reduction. Both xylanase and the enzymatic cocktail (cellulases and laccases) have shown different effects on the structure and the bulk and surface composition of fibers and vessels, more pronounced in the latter. This may be explained by differences in porosity structures affecting the levels of enzymatic attack, and consequently the hemicelluloses and extractives removal due to different mass transfer resistances.
4. Vessel picking and water contact angle: vessel picking counts reduced after enzymatic treatments, hinting an increase of vessel-fiber bond strength after treatment with xylanase, and further increase for the enzymatic cocktail (cellulases and laccase) treated samples. This vessel-fiber bond increase has the potential to reduce the vessel picking phenomena in the printing process. It was also found that fiber sheet samples had lower water contact angle than the vessel rich sheets, supporting the hypothesis that vessels are more hydrophobic than fibers. Xylanase reduced vessels hydrophobicity, with a further increase for the enzymatic complex containing cellulase. Thus, both enzymes reduce vessels hydrophobicity, creating the necessary conditions to minimize the ink refusal phenomena during the printing process.

In summary, vessel and fiber bulk and surface chemical compositions (hemicellulose, lignin, extractives) may explain the differences in swelling, bonding ability, and hydrophobicity of both types of elements. This fact added to their specific porous structures may also explain the different activities shown by the xylanase and the enzymatic cocktail (Supplementary Information).

## Supplementary Information

Supplementary Information.

## Data Availability

The datasets generated during and/or analyzed during the current study are available from the corresponding author on reasonable request. Supplementary data are contained in the Excel file “Supplementary data_Scientific Reports”, and the enzymes data sheets are also provided.

## References

[1] Wiedenhoeft, A. *Structure and Function of Wood*. *Wood Handbook : Wood as an Engineering Material* (U.S. Dept. of Agriculture, Forest Service, Forest Products Laboratory, 2010).

[2] Daniel, G. Wood and fibre morphology. in *Wood Chemistry and Wood Biotechnology*. Vol. 1. 45–70. (Walter de Gruyter GmbH and Co. KG, 2009).

[3] Asikainen, S. *Applicability of Fractionation of Softwood and Hardwood Kraft Pulp and Utilisation of the Applicability of Fractionation of Softwood and Hardwood the Fractions*. (2015).

[4] Dadswell HE, Wardrop AB (1960). Some aspects of wood anatomy in relation to pulping quality and to tree breeding. APPITA.

[5] Alén, R. Structure and chemical composition of wood. in *Forest Products Chemistry* (eds. Stenius, P. & Pakarinen, H.). Vol. 3. 11–57 (Fapet Oy, 2000).

[6] Orblin E, Lindström N, Fardim P (2012). Probing the chemical and surface chemical modification of vessel cell walls during bleaching of eucalyptus pulp. Holzforschung.

[7] Ohsawa, J. Vessel picking in printing papers. in *Tropical Wood Pulp Symposium*. 220–233 (1988).

[8] Jeffries, T. W. Enzymatic treatments of pulps. in *Emerging Technologies for Materials and Chemicals from Biomass* (eds. Rowell, R. M., Schultz, T. P. . & Narayan, R.). Vol. 476. 313–329 (American Chemical Society, 1992).

[9] Bajpai P (1999). Topical paper. Biotechnology.

[10] Cooper III, E. W. *Process for Treating Hardwood Pulp with an Enzyme Mixture to Reduce Vessel Elements Picking.* (1998).

[11] Pathak, P., Kaur, P. & Bhardwaj, N. K. Microbial enzymes for pulp and paper industry: Prospects and developments. in *Microbial Biotechnology: An Interdisciplinary Approach* (ed. Shukla, P.). 163–240. 10.1201/9781315367880 (CRC Press, 2016).

[12] Žnidaršič-Plazl P, Rutar V, Ravnjak D (2009). The effect of enzymatic treatments of pulps on fiber and paper properties. Chem. Biochem. Eng. Q..

[13] Wågberg, L. Chemistry of the fibre surface. in *Paper Chemistry and Technology* (eds. Ek, M., Gellerstedt, G. & Henriksson, G.). 65–92. 10.1515/9783110213447.65 (De Gruyter, 2012).

[14] Laine J, Stenius P, Carlsson G, Ström G (1994). Surface characterization of unbleached kraft pulps by means of ESCA. Cellulose.

[15] Laine J, Stenius P, Carlsson G, Ström G (1996). The effect of ECF and TCF bleaching on the surface chemical composition of kraft pulp as determined by ESCA. Nord. Pulp Pap. Res. J..

[16] Neto CP (2004). Bulk and surface composition of ECF bleached hardwood kraft pulp fibres. Nord. Pulp Pap. Res. J..

[17] Lindström N, Fardim P (2012). Chemistry and surface chemistry of vessels in eucalyptus kraft pulps. O Pap..

[18] Orblin E, Eta V, Fardim P (2011). Surface chemistry of vessel elements by FE-SEM, μ-XPS and ToF-SIMS. Holzforschung.

[19] Atalla RH (1998). Cellulose and the Hemicelluloses: Patterns for the Assembly of Lignin. ACS Symp. Ser..

[20] Atalla RH, Hackney JM, Uhlin I, Thompson NS (1993). Hemicelluloses as structure regulators in the aggregation of native cellulose. Int. J. Biol. Macromol..

[21] Hill CAS, Papadopoulos AN (2001). A review of methods used to determine the size of the cell wall microvoids of wood. J. Inst. Wood Sci..

[22] Lovikka VA, Khanjani P, Väisänen S, Vuorinen T, Maloney TC (2016). Porosity of wood pulp fibers in the wet and highly open dry state. Microporous Mesoporous Mater..

[23] Maloney TC, Paulapuro H (1999). The formation of pores in the cell wall. J. Pulp Pap. Sci..

[24] Maloney TC, Paulapuro H, Stenius P (1998). Hydration and swelling of pulp fibers measured with differential scanning calorimetry. Nord. Pulp Pap. Res. J..

[25] *Certification: The Path for Better Forests—The Newsletter*. http://thenewsletter.pt/2019/02/24/certification-the-path-for-better-forests/. Accessed 9 Dec 2022 (2022).

[26] Sluiter, A. *et al. Determination of Structural Carbohydrates and Lignin in Biomass—NREL/TP-510-42618*. (National Renewable Energy Laboratory, 2008).

[27] Scandinavian Pulp Paper and Board Testing Commitee. *SCAN-CM 65–02 Pulp—Total Acidic Group Content*. (2002).

[28] Chai, X. S., Zhu, J. Y. & Li, J. A simple and rapid method to determine hexeneuronic acid groups in chemical pulps. in *IPST Technical Paper Series* (1999).

[29] Chai XS, Zhu JY, Li J (2001). A simple and rapid method to determine hexeneuronic acid groups in chemical pulps. J. Pulp Pap. Sci..

[30] Nopens M (2020). Determination of mesopores in the wood cell wall at dry and wet state. Sci. Rep..

[31] Thommes M (2015). Physisorption of gases, with special reference to the evaluation of surface area and pore size distribution (IUPAC technical report). Pure Appl. Chem..

[32] Nakamura K, Hatakeyama T, Hatakeyama H (2016). Studies on bound water of cellulose by differential scanning calorimetry. Textile Res. J..

[33] Alves EF, de Oliveira RC, da Silva LHM, Colodette JL (2009). Thermal and spectroscopic analyses on the molecular interaction between eucalyptus kraft pulp components and offset printing inks. Brazil. Arch. Biol. Technol..

[34] Ogata Y (1978). Studies of vessel elements of eucalyptus woods. Part 2. Sheetforming studies and chemical analysis of eucalyptus vessel elements. Japan Tappi J..

[35] Stepanova AV, Oskolski AA, Tilney PM, Van Wyk BE (2013). Wood anatomy of the tribe Podalyrieae (Fabaceae, Papilionoideae): Diversity and evolutionary trends. South Afr. J. Bot..

[36] Luyckx M, Hausman JF, Lutts S, Guerriero G (2017). Silicon and plants: Current knowledge and technological perspectives. Front. Plant Sci..

[37] Raven JA (2003). Cycling silicon—The role of accumulation in plants. New Phytol..

[38] Gellerstedt, G. Analytical methods. in *Wood Chemistry and Wood Biotechnology* (eds. Ek, M., Gellerstedt, G. & Henriksson, G.). 195–218. 10.1515/9783110213409.195 (De Gruyter, 2009).

[39] Fardim P, Gustafsson J, Von Schoultz S, Peltonen J, Holmbom B (2005). Extractives on fiber surfaces investigated by XPS, ToF-SIMS and AFM. Colloids Surf. A Physicochem. Eng. Asp..

[40] Sundberg A, Sundberg K, Lillandt C, Holmbom B (1996). Determination of hemicelluloses and pectins in wood and pulp fibres by acid methanolysis and gas chromatography. Nord. Pulp Pap. Res. J..

[41] Holmbom, B. & Stenius, P. Analytical methods. in *Papermaking Science and Technology 3: Forest Products Chemistry* (ed. Stenius, P.). Vol. 3. 62–78 (2000).

[42] Aksenov AS (2020). Biocatalysis of industrial kraft pulps: Similarities and differences between hardwood and softwood pulps in hydrolysis by enzyme complex of *Penicillium verruculosum*. Catalysts.

